# *Helicobacter pylori* and Gastric Cancer: Pathogenetic Mechanisms

**DOI:** 10.3390/ijms24032895

**Published:** 2023-02-02

**Authors:** Silvia Salvatori, Irene Marafini, Federica Laudisi, Giovanni Monteleone, Carmine Stolfi

**Affiliations:** 1 Department of Systems Medicine, University of Rome “Tor Vergata”, 00133 Rome, Italy; 2 Gastroenterology Unit, Policlinico Universitario Tor Vergata, 00133 Rome, Italy

**Keywords:** inflammation, cytokines, ROS, virulence factors, CagA, genomic instability, MMR

## Abstract

Gastric cancer is the sixth most commonly diagnosed cancer and the fourth leading cause of cancer death worldwide. *Helicobacter pylori* (*H. pylori*) is one of the main risk factors for this type of neoplasia. Carcinogenetic mechanisms associated with *H. pylori* are based, on the one hand, on the onset of chronic inflammation and, on the other hand, on bacterial-specific virulence factors that can damage the DNA of gastric epithelial cells and promote genomic instability. Here, we review and discuss the major pathogenetic mechanisms by which *H. pylori* infection contributes to the onset and development of gastric cancer.

## 1. Introduction

There were over 1,000,000 new cases of gastric cancer in 2020 and it was responsible for approximately 768,000 deaths, making it the sixth most frequently diagnosed cancer and the fourth leading cause of cancer death [1]. Incidence rates are twice as high in men than in women and remarkably high in East Asia [1]. According to the American Cancer Society, most cases of gastric cancer (about 90–95%) are adenocarcinomas, arising in gland cells that produce mucus and stomach juices. Other less frequent types of gastric neoplasms include: (i) lymphomas that start in the lymph tissues of the stomach [e.g., mucosa-associated lymphoid tissue (MALT) lymphoma], accounting for approximately 4% of all gastric cancers; (ii) gastrointestinal stromal tumors, or GISTs, a rare type of gastric cancer (less than 1%) that forms in a special cell found in the lining of the stomach called interstitial cells of Cajal; (iii) carcinoid tumors, which typically start in the hormone-producing cells of the stomach and account for about 3% of gastric cancer incidence; and (iv) hereditary diffuse gastric cancer (HDGC) (about 1% to 3%). Unless otherwise noted, the articles and the molecular mechanisms described here refer to gastric adenocarcinomas.

**Figure 1:**
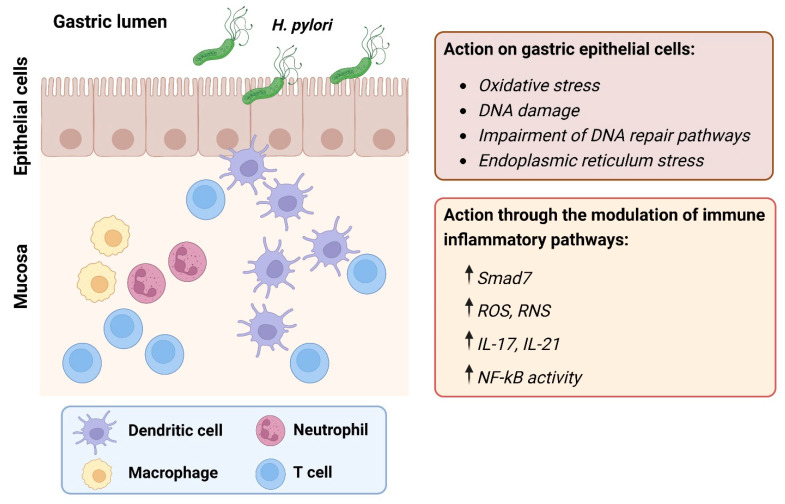
Direct and indirect actions of *Helicobacter pylori* (*H. pylori*) on gastric epithelial cells during gastric carcinogenesis. *Abbreviations*: Smad7: suppressor of mothers against decapentaplegic 7; ROS: reactive oxygen species; RNS: reactive nitrogen species; IL: interleukin; NF-ĸB: nuclear factor kappa-light-chain-enhancer of activated B cells. Created with Biorender.com.

*Helicobacter pylori* (*H. pylori*) is the main risk factor for gastric cancer, along with alcohol consumption and smoking. Other dietary habits associated with an increased risk of developing the disease are the consumption of salt-preserved foods and a low-fruit diet. [2]. *H. pylori* is a gram-negative, helix-shaped, microaerophilic bacterium that specifically colonizes the gastric epithelium causing the most common bacterial infection in the world [3]. Although most patients bearing an *H. pylori* infection are asymptomatic, all will sooner or later develop gastritis which, in the long run, may result in the emergence of gastroduodenal ulcers and eventually gastric carcinoma and lymphoma of the lymphoid tissue associated with the mucosa [4]. Although the reasons for such heterogeneous responses are not known, clues point toward the involvement of both bacterial and host factors [4] (Figure 1).

In particular, it is known that *H. pylori* infection induces chronic non-atrophic gastritis that can progress to intestinal metaplasia, dysplasia, and finally gastric cancer [5]. 

In this sequential process, both the chronic inflammatory response and the presence of specific bacterial virulence factors, especially the cytotoxin-associated gene A (CagA) and vacuolating cytotoxin A (VacA), play a fundamental role in causing damage to host cell DNA and in the activation of specific pathways that sustain cell survival, with both processes often supporting each other [5]. *H. pylori* infection results in a reduced ability of the infected cells to repair DNA damage [6]. Such an effect may increase genetic instability and progressively lead to the accumulation of mutations that, in turn, can activate oncogenes and deactivate tumor suppressor genes, with the ultimate result of increasing the risk of developing gastric cancer over time [6,7]. 

In this article, we review and discuss the major pathogenetic mechanisms that link *H. pylori* infection with gastric carcinogenesis. The studies summarized here were retrieved from the biomedical literature using ”H. pylori” AND “gastric cancer” and ”H. pylori” AND “carcinogenesis” from PubMed, Scopus, and Web of Science bibliographic databases. Studies were selected after applying the following inclusion and exclusion criteria. The inclusion criteria were: (1) articles published in the English language and within the past 25 years; and (2) articles describing the main mechanisms linking *H. pylori* with gastric carcinogenesis with a high citation rate. The exclusion criteria were: (1) articles published in languages other than English; and (2) articles not available in full-text format.

## 2. Role of Chronic Inflammation

Chronic inflammation contributes to the pathogenesis of several types of malignant diseases [8] and is particularly relevant for *H. pylori*-related gastric tumorigenesis [9]. *H. pylori* infection leads to inflammatory reactions through a variety of molecular pathways induced in both gastric epithelial cells, which the bacterium first contacts, and in circulating immune cells recruited to the site of infection, such as neutrophils, macrophages, and lymphocytes [10].

### 2.1. Innate Immune Response and Oxidative Stress

Among the immune cells that participate in the innate immune response, neutrophils actively seek, ingest, and destroy pathogenic microorganisms, this latter action realized through the production of substances with antimicrobial activity (such as oxidants, proteinases, and antimicrobial peptides) [11]. In particular, among the oxidants produced by neutrophils, reactive oxygen species (ROS) and reactive nitrogen species (RNS) exert a dual function. On the one hand, ROS and RNS work as antimicrobial agents due to their ability to directly kill microbial pathogens. On the other hand, they participate, as signaling molecules, in the regulation of physiological functions of neutrophils through the modulation of different molecular cascades [12]. However, in the gastric mucosa infected by *H. pylori*, neither ROS or RNS eliminate the bacterium. Indeed, *H. pylori* may survive oxidative stress due to defense mechanisms related to the production of antioxidant enzymes (e.g., NapA, catalase, superoxide catalase) [13]. Interestingly, *H. pylori* may contribute to increased oxidative stress in gastric epithelial cells [14] (Table 1).

*H. pylori*-derived CagA induces the expression of spermine oxidase (SMOX), an enzyme for the back-conversion of spermine to spermidine. This reaction leads to the production of H_2_O_2_ as a by-product. Increased H_2_O_2_ may, in turn, cause ROS accumulation through mitochondrial membrane depolarization, as well as activation of caspase-mediated apoptosis [15].

Overproduction of ROS and RNS induces various types of DNA damage, including point mutations, DNA adducts, and single- or double-strand DNA breaks (DSB) [13]. Among these, 8-hydroxydeoxyguanosine (8-OHdG), which is the main oxidatively modified product of DNA, is significantly expressed in gastric cancer tissues [20]. Apurinic/apyrimidinic endonuclease/redox factor 1 (APE1) is one of the main regulators of the cellular response to oxidative stress and is involved in the transcriptional regulation of gene expression during the adaptive cellular response to oxidative stress and the base excision repair system [21]. *H. pylori* infection can differently regulate APE1 function. While the *H. pylori*-driven increase in oxidative stress upregulates APE1 levels, helping repair DNA damage, a chronic *H. pylori* infection can eventually inhibit the expression of APE1 leading to genetic instability [22].

### 2.2. Adaptive Immune Response

The molecular pathways controlling adaptive immune responses driven by *H. pylori* are complex, but locally induced cytokines seem to play a key role in maintaining the ongoing inflammation. *H. pylori* infection is associated with marked mucosal induction of T helper (Th) type 1 (e.g., IFN-γ) and Th17-type (e.g., IL-17A, IL-21) cytokines that are governed by specific antigen-presenting cell-derived molecules, such as interleukin IL-12 and IL-23 [23]. 

D’ Elios and colleagues provided evidence for a local production of anti-*H. pylori* IgA and IgG and, in particular, for a specific response of Th1 effectors—resulting in increased synthesis of interferon (IFN)-γ, tumor necrosis factor (TNF)-α, and IL-12—in the gastric antrum of *H. pylori*-infected patients, which can play a role in the genesis of either peptic ulcer or *H. pylori*-related gastric B cell lymphoma [16]. Notably, the emergence of serum anti-*H. pylori* IgG and IgA may indicate a widespread immune response caused by bacterial infection and serve as a highly accurate, simple, and non-invasive method for monitoring *H. pylori* infection status in patients with gastric atrophy, intestinal metaplasia, and dysplasia [24].

Transforming growth factor (TGF)-β1 constitutes a key negative regulator of Th1-type immune responses [25]. In Crohn′s disease, one of the main inflammatory bowel diseases in humans traditionally associated with a Th1 cytokine profile, the TGF-β1 pathway is compromised due to the high expression of Mothers against decapentaplegic homolog (Smad)7, which negatively regulates TGF-β1-associated Smad signaling through various mechanisms (e.g., inhibition of Smad 2/3 phosphorylation, inactivation/degradation of TGF-β1 receptors) [26]. Elevated expression of Smad7, as well as defective Smad 2/3 phosphorylation, were reported in both whole biopsy samples and immune cells isolated from the inflamed gastric tissue of *H. pylori*-infected patients, as compared with specimens/cells isolated from the stomach of patients without *H. pylori* infection [17]. In the former, inhibition of Smad7 through a specific antisense oligonucleotide restored the TGF-β1 regulatory cascade and concomitantly reduced the levels of IFN-γ and T-bet, two hallmarks of Th1 immune response. Notably, Smad7 was inducible by IFN-γ in normal gastric biopsies via a STAT1-dependent mechanism, and neutralization of IFN-γ in biopsy samples from *H. pylori*-infected patients reduced Smad7 expression. Overall, these data suggest that, in the gastric mucosa infected with *H. pylori*, IFN-γ triggers the upregulation of Smad7 that, in turn, prevents endogenous TGF-β1 from dampening the ongoing tissue-damaging Th1 response [17]. T cell-derived cytokines have been reported to enhance the synthesis of matrix metalloproteinases (MMP), thus contributing to mucosal ulceration and epithelial damage. In particular, IL-21 is constitutively expressed in the gastric mucosa and higher levels of the cytokine were found in gastric biopsies and purified mucosal T cells taken from *H. pylori*-infected patients compared to those taken from normal patients and disease controls [18]. The human gastric adenocarcinoma cell line AGS responded to IL-21 by increasing the production of MMP-2 and MMP-9 in a NF-kB-dependent fashion and treatment of *H. pylori*-infected gastric explants with an antibody against IL-21 decreased the production of epithelial cell-derived MMP-2 and MMP-9 [18]. Another cytokine overproduced in the *H. pylori*-infected gastric mucosa is IL-17A [19], which is positively correlated with gastric cancer progression and invasiveness [19,27]. During *H. pylori* infection, IL-17A is produced by T lymphocytes of the lamina propria and non-T cells through a process regulated by IL-1β, IL-21, and IL-23 [28]. IL-17A has been reported to stimulate both immune and non-immune cells to produce multiple inflammatory mediators, such as IL-1β, IL-6, TNF-α, and MMPs, thus perpetuating mucosal inflammation and degradation [23].

Despite inducing an inflammatory response, *H. pylori* can persist in the gastric mucosa for decades. Recent studies have shown that *H. pylori*’s expression of cholesterol-α-glucosyltransferase reduces cholesterol levels in infected gastric epithelial cells by blocking the IFN-γ signaling pathways, allowing bacteria to escape the host′s inflammatory response [29].

## 3. *H. pylori* Virulence Factors

**Figure 2:**
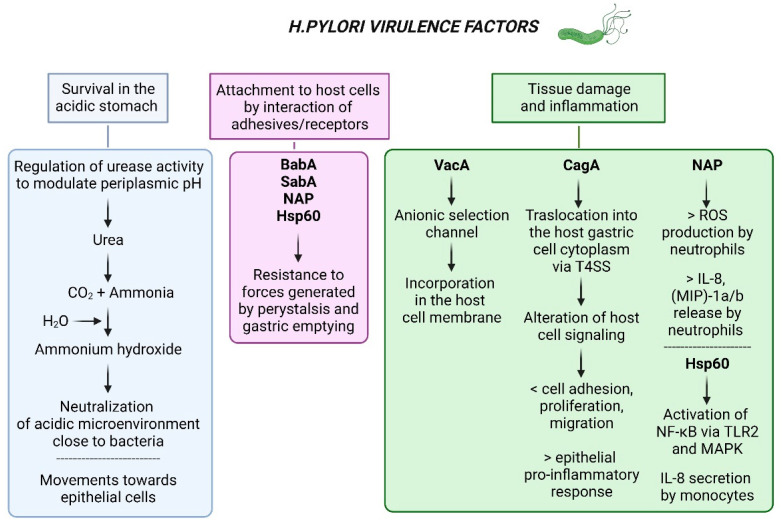
Virulence factors involved in the pathogenicity of *H. pylori*. *Abbreviations*: BabA: blood group antigen-binding adhesion A; SabA: sialic acid-binding adhesion A; NAP: neutrophil activating protein; Hsp: heat shock protein; VacA: vacuolating cytotoxin gene A; CagA: cytotoxin-associated gene A; T4SS: type IV secretion system; NAP: neutrophil-activating protein; ROS: reactive oxygen species; IL: interleukin; MIP: macrophage inflammatory protein; NF-kB: nuclear factor kappa-light-chain-enhancer of activated B cells; TLR: toll-like receptor; MAPK: mitogen-activated protein kinase. Created with Biorender.com.

Once in the host stomach, four steps are critical for *H. pylori* to establish successful colonization and persistent infection: (i) survival in the acidic stomach environment; (ii) movement towards epithelium cells through flagella-mediated motility; (iii) attachment to host cells through interaction of microbial adhesins with host cell receptors; and (iv) inducing tissue damage by toxins release [30] (Figure 2). 

*H. pylori* survives in the acidic environment of the stomach thanks to a mechanism that allows the bacterium to modulate the periplasmic pH through the regulation of urease activity. The *urease* gene cluster is composed of seven genes, including catalytic subunits (i.e., *ureA/B*), an acid-gated urea channel (*ureI*), and accessory assembly proteins (*ureE-H*) [31]. The intrabacterial urease activity is required for acid resistance by *H. pylori*, and this activity is regulated by the UreI channel with proton urea, which allows entry of urea only under acidic conditions to prevent lethal alkalinization during periods of relative neutrality. Extracellular urease breaks down urea into carbon dioxide and ammonia, which, when combined with water, determines the production of ammonium hydroxide. Therefore, *H. pylori* can pass through gastric juice safely as ammonium hydroxide neutralizes the acidic microenvironment close to bacteria [32]. *H. pylori* moves to the basal layer of the gastric epithelium, where the pH value is close to 7.0, per 4–7 polar flagella action. Kao et al. reported that patients infected with *H. pylori* strains with increased motility showed a higher bacterial density, resulting in a more severe inflammatory response in the upper part of the stomach as well as pathological results. In these respects, the flagellum can be considered a colonization and virulence factor in the initial phase [33]. The interaction of bacterial adhesins with host cell receptors protects bacteria from displacement by forces such as those generated by peristalsis and gastric emptying. Additionally, bacteria obtain metabolic substrates and nutrients to improve growth by releasing toxins to damage host cells. Although blood-antigen binding protein A (BabA) and sialic acid-binding adhesin (SabA) are the well-characterized adhesins studied so far, several other adhesins in *H. pylori* are known to adapt to different hosts/tissues, including in particular neutrophil activating protein (NAP) and heat shock protein 60 (Hsp60) [34]. As already mentioned, NAP can stimulate a high production of oxygen radicals by neutrophils, leading to damage to local tissues. Furthermore, NAP induces the expression and release of IL-8, the macrophage inflammatory protein (MIP)-1a, and MIP-1b by neutrophils. As a result, NAP is strongly associated with neutrophil and mononuclear cell infiltration into the gastric mucosa after *H. pylori* infection [35]. Hsp60 has been identified as one of the immunogenic potentials of the bacterium. Hsp60 induces the activation of NF-kB through TLR2 and the mitogen-activated protein (MAP) kinase pathway and, therefore, induces human monocytes to secrete IL-8 [36]. In addition, anti-Hsp60 antibodies are constantly detected in *H. pylori*-infected patients and the titers are associated with the progression of gastritis or gastric cancer [37].

### 3.1. Cytotoxin-Associated Gene A (CagA) and the Cytotoxin-Associated Gene Pathogenicity Island (cagPAI)

Several *H. pylori* virulence factors have been identified so far [38]. Among these, CagA and the cagPAI play a central role in the pathogenesis of diseases associated with *H. pylori* (e.g., acute gastritis, gastric ulcer) as well as in the development of gastric cancer [39]. The epidemiological prevalence of *H. pylori* positive for CagA in Western countries is almost 60% and around 90% in Asian countries [40]. The *cagPAI* consists of 40 kb chromosomal DNA and contains more than 30 genes. Among them, *cagPAI* carries at least six genes that are homologous to the type IV secretion system (T4SS), acting as a molecular syringe that injects the bacterial protein CagA into the cytoplasm of the host gastric cell [41]. The translocated CagA protein localizes to the inner surface of the plasma membrane through interactions with phosphatidylserine. Once injected into the cytoplasm via T4SS, CagA can alter host cell signaling in both a phosphorylation-dependent and -independent manner.

Phosphorylated CagA binds to the phosphatase SHP-2 and affects cell adhesion, spread, and migration [42]. Moreover, CagA can also affect the host cell behavior in additional ways, such as by inducing cytoskeleton rearrangements, affecting cell proliferation, and by stimulating gastric epithelial cells to secrete IL-8 [39].

CagA also exerts phosphorylation-independent actions, many of which remain unclear. Suzuki and colleagues identified a conserved motif in the C-terminus of nonphosphorylated CagA designated as CRPIA (conserved repeat responsible for phosphorylation-independent activity) [43]. The authors showed that the CRPIA motif in nonphosphorylated CagA participated in the interaction with the activated hepatocyte growth factor receptor Met in the host cells, leading to the persistent activation of the phosphatidylinositol 3-kinase/Akt pathway. This effect contributed to the promotion of gastric cell proliferation and the establishment of a pro-inflammatory microenvironment associated with the development of chronic gastritis and gastric cancer through the activation of NF-κB and β-catenin signaling, respectively [43].

Aberrant hypermethylation of the promoter CpG islands of tumor suppressor genes, leading to their inactivation, occurs at high levels during gastric inflammation and carcinogenesis [44]. In this context, Zhang et al. reported that *H. pylori* CagA increased tumor suppressor gene hypermethylation by upregulating the expression of DNMT1, a methyltransferase involved in the malignant transformation of various cancers [45], via the AKT–NF-κB pathway [46]. Mechanistically, unphosphorylated CagA increased AKT phosphorylation by enhancing its interaction with 3-phosphoinositide-dependent protein kinase-1 (PDK1) in the host cytoplasm, which then resulted in DNMT1 overexpression via NF-κB activation. This increased methylation activity led to hypermethylation and inactivation of the tumor suppressor gene O-6-methylguanine-DNA methyltransferase (MGMT) in stomach epithelial cells that can contribute to the development of gastric cancer [46].

The mammalian Hippo tumor suppressor signaling pathway is crucial in maintaining the size and homeostasis of developing organs [47]. Yes-Associated Protein (YAP), a key downstream effector of the Hippo molecular cascade, is involved in specific cellular functionalities that control gastric epithelial cell proliferation, differentiation, and migration [47]. Increased activation/expression of YAP is functionally important for proliferative and pro-survival activity in cancer cells and is positively associated with the progression of a variety of human cancers [48]. Li and co-workers performed elegant experiments to demonstrate that *H. pylori*-mediated delivery of CagA into cultured gastric epithelial cells promotes the oncogenic YAP pathway, resulting in the downregulation of E-cadherin and an increase in the epithelial mesenchymal transition program, further promoting gastric carcinogenesis [49].

### 3.2. Vacuolating Cytotoxin A (VacA)

Another key *H. pylori*-related virulence factor is the VacA, considered a multifunctional toxin responsible for eliciting multiple effects on host cells, including vacuolization, necrosis, and apoptosis. The VacA complex can also be incorporated into the host cell membrane and present the characteristic of an anionic selection channel. Acting as a channel, VacA can promote the release of bicarbonate and organic anions into the host cytoplasm, thus helping *H. pylori* colonization through the outflow of potential metabolic substrates for bacterial growth [50]. Previous studies also indicated that exogenous VacA may target different organelles of the host cells. In fact, the VacA complex may enter the endosome through endocytosis [51] and extracellularly applied VacA was supposed to target mitochondria, as it was shown to induce the release of cytochrome C and apoptosis [52]. Furthermore, VacA was found to trigger endoplasmic reticulum (ER) stress response to activate autophagy and induce apoptosis [53,54]. *H. pylori*-mediated induction of apoptosis in gastric epithelial cells may play a role not only in gastric injury but also in the development of gastric atrophy and cancer.

## 4. Genomic Instability

We have seen that *H. pylori* infection causes inflammation and leads both directly and indirectly to DNA damage, such as oxidative damage and DSBs, in host cells. Furthermore, the resulting genetic and/or epigenetic disturbances alter the choice of DNA repair paths. These changes cause inaccurate DNA repair, genomic instability, and chromosomal aberration, which may ultimately promote gastric carcinogenesis. DNA damage repair can be achieved through a large number of proteins, which include several important DNA repair pathways, such as mismatch repair (MMR), base excision repair (BER), nucleotide excision repair (NER), homologous recombination (HR), non-homologous end join (NHEJ), and alternative end join (A-EJ) [55]. Such DNA repair pathways are complemented by a series of signaling reactions that ultimately stop the cell cycle and promote cell death in the event of irreparable damage. Many studies have shown that *H. pylori* alters the expression of DNA repair genes and/or interferes with the activity of DNA repair [56].

### 4.1. H. pylori Infection and the MMR Pathway

The MMR pathway is one of the most studied DNA repair mechanisms. The MMR machinery maintains the stability of the genome by correcting errors in newly synthesized strains during DNA replication. Diseases caused by MMR defects include brain tumors and hereditary non-polyposis colorectal cancer [57,58,59].

Several human MMR proteins have been identified in human cells. Base–base mismatches and small insertion–deletion loops are preferentially recognized by the hMSH2–hMSH6 (hMutSα) complex; whereas MutSβ, combining the MSH2 and MSH3 proteins, the hMSH2–hMSH3 (hMutSβ) complex, mainly binds to larger insertion–deletion loops [60]. Both complexes are required for MMR. Additionally, four human MutL homologs (hMLH1, hMLH3, hPMS1, and hPMS2) have been identified. hMLH1 heterodimerizes with hPMS2, hPMS1, or hMLH3 to form hMutLα, hMutLβ, or hMutLγ, respectively. While hMutLα is required for MMR, the role of the other two in this process is poorly understood [61,62].

In eukaryotic cells, DNA MMR defects can be detected as instability in simple sequence repeats or short tandem repeats called microsatellites. Therefore, microsatellite instability (MSI) is considered a hallmark of MMR deficiencies [59,60]. MSI has been indicated as a reliable biomarker of stomach cancer [63]. It should be noted that patients with MSI-positive gastric cancers exhibit more active *H. pylori* infections than those with MSI-negative ones, suggesting that *H. pylori* itself may affect the DNA MMR system during stomach carcinogenesis [64] (Table 2). 

Additionally, MSI was found in gastric intestinal metaplasia of patients with or without gastric carcinoma, implying that the accumulation of MSI in areas of intestinal metaplasia may contribute to the development of gastric cancer [64].

Kim and co-workers investigated whether *H. pylori* and related products could affect the MMR pathway in gastric cancer cell lines. The authors found a decrease in the level of expression of the MLH1, PMS1, PMS2, MSH2, and MSH6 proteins after *H. pylori* infection. Interestingly, this decrease was independent of CagA virulence factor and was correlated with downregulation of MSH2 and MSH6, but not of MLH1 mRNA transcripts. Of note, the expression of MLH1 and MSH2 in cells that underwent *H. pylori* eradication returned to values similar to those reported in uninfected cells, indicating a reversible inhibition of MMR gene expression [65].

In line with these findings was the work of Park et al., who examined the expression of MLH1 and MSH2 in patients with chronic *H. pylori* infection before and after eradication treatment [66]. The authors reported that bacterial eradication increased the expression of MLH1 and MSH2 compared to samples taken before the treatment, suggesting that chronic *H. pylori* infection may negatively affect the MMR machinery in the gastric epithelium, thus increasing the risk of mutation accumulation. This hypothesis was supported by studies on human gastric tissue samples performed by Mirzaee and colleagues, showing that the percentage of MLH1-positive epithelial cell nuclei was lower in *H. pylori*-infected patients compared to noninfected individuals [67].

To outline the biological role of the *H. pylori*-driven decrease in MMR expression, Machado et al. examined the effect of the bacterial infection on the main repair pathways of gastric epithelial cells in vitro and in vivo [68]. Following *H. pylori* infection, the expression of MMR-related genes and proteins, as well as cellular MMR activity, declined in both AGS cells and in the gastric cells of C57BL/6 mice. It is noteworthy that this decrease did not rely on the virulence factors of the bacteria. The authors also showed that bacterial infection decreased overall MMR activity and increased the risk of accumulating mutations in gastric epithelial cells, thus promoting genetic instability [68].

Taken together, these studies highlight a key role for *H. pylori* infection in the onset of MMR deficiency in gastric epithelial cells.

### 4.2. H. pylori Infection and the BER Pathway

Base Excision Repair (BER) is another important repair pathway that is key to ensuring genome stability, as it repairs a number of endogenously generated lesions in the DNA double-helix structure, such as oxidation, deamination, methylation, depurination, and hydroxylation. BER is initiated by recognition and cleavage of the damaged base by specific DNA glycosylases, resulting in the creation of apurinic/apyrimidinic (AP) sites [73,74]. The generated AP sites are cytotoxic and mutagenic and, therefore, further processed by DNA glycosylases with AP-lyase activity (e.g., OGG1), whose turnover is stimulated by APE1 [75,76]. After processing of the AP site, the single nucleotide gap is filled by Pol beta and the nick is sealed by DNA ligase I to complete the repair reaction [74]. 

Machado et al. showed that APE1, but not OGG1 expression, was downregulated in gastric cells infected with *H. pylori* [68]. These results suggest that *H. pylori* infection causes an imbalance between the generation and repair of AP sites, which is highly mutagenic [77]. In apparent contrast with this finding, other groups have reported an increase in APE1 levels in both cultured cells and gastric epithelial cells isolated from mucosal biopsies during *H. pylori* infection [22,69]. These opposite results could rely on not superimposable experimental conditions, such as a different multiplicity and time of infection [69], and the use of *H. pylori* protein extracts instead of live bacteria [22]. In patients with *H. pylori*-positive gastritis, APE1 levels were also upregulated, but decreased after bacterial eradication [22].

Overall, these results indicate that *H. pylori* infection may differently affect the BER pathway through different mechanisms, leading to different outcomes of DNA damage in the host cells.

### 4.3. H. pylori Infection and other Repair Pathways

A combination of decreased expression of other repair genes together with an increased level of genotoxic factors could enhance the accumulation of somatic mutations in gastric epithelial cells, thus favoring a malignant transformation. Toller and colleagues showed that DSBs accumulate in a time- and dose-dependent manner in both gastric cell lines and primary gastric epithelial cells that were incubated with *H. pylori*. In particular, DSBs appeared at mitosis and required direct contact between living bacteria and host cells, but not the *H. pylori* virulence factor VacA or ROS-mediated DNA damage [78]. RNA-seq and microarray analyses of gastric cells infected by *H. pylori* showed that bacterial infection downregulated some components of the HR machinery (e.g., ATR, ATRIP, RAD51, RPA1, MRE11, NBS1), thus suggesting a shift in the choice of the DNA repair pathway away from HR [70,79]. Hartung et al. demonstrated that, upon *H. pylori* infection, DSBs are introduced by NER via the endonucleases XPG and XPF in a T4SS-dependent fashion [71]. Interestingly, *H. pylori*-induced DSBs were repaired via error-prone NHEJ rather than HR, possibly due to the upregulation of NHEJ-related genes and the downregulation of HR-related genes [71].

In line with this latter result is a recent work by Han and co-workers [72]. The authors found that the long noncoding RNA SNHG17 was upregulated by *H. pylori* infection and significantly increased the percentage of DSBs. Nuclear SNHG17-mediated recruitment of NONO, an RNA- and DNA-binding nuclear factor involved in the NHEJ pathway, together with the role of cytoplasmic SNHG17 as a decoy for miR-3909, which regulates RAD51 expression, shifted the DSB repair balance from HR toward NHEJ. Notably, the elimination of SNHG17 in gastric cancer cells inhibited chromosomal aberrations after *H. pylori* infection and overexpression of SNHG17 was correlated with a poor prognosis in patients with gastric cancer [72].

Taken together, these findings indicate that various modifications of DNA repair functions are closely associated with DSB formation and chromosomal abnormalities during the development of *H. pylori*-related gastric cancers.

## 5. Conclusions

The etiology of gastric cancer is complex and multifactorial, involving environmental and host-related factors as well as genetic and epigenetic alterations. *H. pylori* infection has been shown to call for multiple known mechanisms (and probably others still to be discovered) to induce the onset and progression of gastric cancer, making it the most important risk factor in the pathogenesis of this neoplasia. In addition to efforts to discover and validate new biomarkers [80], and changes in people’s lifestyle and dietary habits, prevention strategies must therefore be adopted to identify high-risk patients and offer a personalized therapy before the start of precancerous lesions. In this context, it is worth noting that a recent prospective, randomized, placebo-controlled trial with 26.5 years of follow-up provided strong evidence that *H. pylori* eradication therapy could confer long-term protection against gastric cancer in high-risk populations, especially for infected individuals without advanced gastric lesions at baseline [81].

## Figures and Tables

**Table 1:** Effects of *H. pylori* infection on tumor-promoting chronic inflammation.

Category	Target	Effects	Disease Course	Ref
Oxidative stress	Neutrophils/macrophages	Increased production of ROS and RNS.	Onset	[14]
	SMOX	Increased production of H_2_O_2_ as a by-product of conversion of spermine to spermidine.	Onset	[15]
Adaptive immune response	Th1/Th17 cells	Increased synthesis of IFN-γ, TNF-α, and IL-12.	Onset, development	[16]
	SMAD7	Prevents endogenous TGF-β1 from dampening the ongoing tissue-damaging Th1 response.	Development	[17]
	IL-21	Increased production of MMP-2 and MMP-9 in a NF-ĸB-dependent fashion.	Development	[18]
	IL-17A	Increased production of IL-1β, IL-6, TNF-α, and MMPs.	Development	[19]

Abbreviations: ROS: reactive oxygen species; RNS: reactive nitrogen species; SMOX: spermine oxidase; Th1: T helper type 1; Th17: T helper type 17; IFN-γ: interferon- γ; TNF-α: tumor necrosis factor-α; IL: interleukin; Smad7: suppressor of mothers against decapentaplegic 7; TGF-β1: transforming growth factor-β1; MMP: matrix metalloproteinase; NF-ĸB: nuclear factor kappa-light-chain-enhancer of activated B cells.

**Table 2:** Effects of *H. pylori* infection on DNA repair machineries.

Repair Pathway	Target	*H. pylori*–Associated Events	Disease Course	Ref
MMR	MLH1, PMS1, PMS2, MSH2, and MSH6	Decreased level following *H. pylori* infection	Onset, development	[65]
	MLH1 and MSH2	Increased expression after *H. pylori* eradication	Onset, development	[65,66]
	MLH1	Decreased fraction of MLH1-positive epithelial cell nuclei in *H. pylori*-infected patients	Onset, development	[67]
	MMR genes	Decreased expression and activity after *H. pylori* infection both in vitro and in vivo	Onset, development	[68]
BER	APE1	Decreased expression after *H. pylori* infection resulting in an imbalance between the generation and repair of AP sites, which is highly mutagenic	Onset, development	[68]
	APE1	Increased levels in both cultured cells and in primary gastric epithelial cells during *H. pylori* infection	/	[22]
	APE1	Upregulation during *H. pylori* infection, downregulation after bacterial eradication	/	[69]
HR and NHEJ	ATR, ATRIP, RAD51, RPA1, MRE11, and NBS1	Decreased expression following *H. pylori* infection	Onset, development	[70]
	NHEJ-related genes	Increased expression after *H. pylori* infection	Onset, development	[71]
	SNHG17	Shifting of the DSB repair balance from HR toward NHEJ	Onset, development	[72]

Abbreviations: MMR: mismatch repair; MLH1: MutL homolog 1; PMS1 and 2: PMS1 homolog 1 and 2; MSH: MutS homolog; APE1: apurinic/apyrimidinic endonuclease/redox factor 1; HR: homologous recombination; NHEJ: non-homologous end joining; ATR: ataxia telangiectasia and Rad3-related protein; ATRIP: ATR-interacting protein; RAD51: RAD51 recombinase; RPA1: replication protein A1; MRE11: MRE11 homolog, double strand break repair nuclease; NBS1: Nijmegen breakage syndrome 1; SNHG17: small nucleolar RNA host gene 17.

## Data Availability

No new data were created or analyzed in this study. Data sharing is not applicable to this article.
